# Scientific Evidence for Different Options for GDM Screening and Management: Controversies and Review of the Literature

**DOI:** 10.1155/2017/2746471

**Published:** 2017-04-10

**Authors:** Claudia Caissutti, Vincenzo Berghella

**Affiliations:** ^1^Department of Experimental Clinical and Medical Science, DISM, Clinic of Obstetrics and Gynecology, University of Udine, Udine, Italy; ^2^Division of Maternal-Fetal Medicine, Department of Obstetrics and Gynecology, Sidney Kimmel Medical College, Thomas Jefferson University, Philadelphia, PA, USA

## Abstract

*Background. *Gestational diabetes (GDM) affects up to 7% of pregnant women and is associated with several maternal and perinatal morbidities. International organizations suggest several different recommendations regarding how to screen and to manage GDM.* Objective. *We aimed to analyze the most important and employed guidelines about screening and management of GDM and we investigated existing related literature.* Results. *We found several different criteria for screening for GDM, for monitoring GDM, and for starting pharmacological therapy. When using IADPSG criteria, GDM rate increased, perinatal outcomes improved, and screening became cost-effective. Compared to no treatment, treatment of women meeting criteria for GDM by IADPSG criteria but not by other less strict criteria has limited evidence for an effect on adverse pregnancy outcomes.

## 1. Introduction

Gestational diabetes (GDM) can be broadly defined as glucose intolerance during pregnancy that affects women without previous diagnosis of diabetes or unknown state. The incidence is about 7% worldwide and this rate has been growing during the last decades and is estimated to increase in the future. The most important risk factors are maternal overweight and obesity, age greater than or equal to 35 years at delivery, hypertension, metabolic syndrome, nonwhite ethnicity, family history of diabetes mellitus, prior unexplained stillbirth, prior infant with congenital anomaly (if not screened during that pregnancy), prior macrosomic infant, history of gestational diabetes, chronic use of steroids, glycosuria, and known impaired glucose metabolism [1].

The importance of GDM is linked to the consequences of pregnancy and also after pregnancy to both mother and newborn. Hyperglycemia in the mother causes abnormal metabolism while in the fetus it causes hyperinsulinemia and its consequences, and incidence of complications is inversely proportional to glucose control. Macrosomia, polyhydramnios, operative delivery, shoulder dystocia, birth injury, perinatal mortality, hypertensive disorders and preeclampsia, congenital malformations (OR: 1.2–1.4), and risk of cesarean delivery are higher in women with GDM; in the long term, women with GDM have a higher risk of developing type 2 diabetes mellitus and cardiovascular diseases; long-term sequelae for offspring are obesity and metabolic syndrome. Approximately 50% of women identified as having GDM will develop frank diabetes within 10 years [2].

To prevent or decrease the risk of GDM, weight loss before pregnancy and cardiovascular exercise could be useful. In fact, aerobic exercise for 35–90 minutes 3-4 times per week during pregnancy is associated with a significantly higher incidence of vaginal delivery and a significantly lower incidence of cesarean delivery, with a significantly lower incidence of gestational diabetes mellitus and hypertensive disorders [3]. Prompt diagnosis and management are important to reduce worse pregnancy outcomes.

Nonetheless, screening, management, and follow-up of GDM are controversial on international organizations recommendations.

## 2. Screening Controversies

The aim of screening is to identify asymptomatic pregnant women at high risk of developing GDM. Screening appears to be cost-effective for prevention of obstetrical adverse outcomes and long-term consequences of GDM [4].

Regarding the effect of screening [1] on obstetrical outcomes, there are many controversies:

- Indications for screening (who): universal versus selective screening
- Timing of screening (when): early screening versus at 24–28 weeks
- Type of screening (how): One- versus Two-Step
- Criteria for diagnosis: recommendations of international organizations are not standardized

(a) The* population to screen* has not been uniformly identified. There are two possible approaches.

*(i) Selective Screening*. Only women with risk factor for GDM are offered to be screened, that is, age > 25 years; ethnic origin Hispanic, African, Native American, South or East Asian, or Pacific Islander; BMI > 25; previous personal or family history of impaired glucose tolerance; or history of adverse obstetric outcomes associated with GDM.

*(ii) Universal Screening*. All women are subjected to screening; in developed countries where overweight and obesity are widespread health problems, this could be the best choice to avoid undiagnosed GDM.

Universal screening is the most commonly adopted method in the USA, while in other countries such as Italy the selective approach is preferred [5].

(b) When identifying the population, it is essential to decide the right* time to screen*.

Women with risk factors and high suspicion of undiagnosed type 2 DM (i.e., obesity, metabolic syndrome) should be screened before pregnancy or at the first prenatal visit (early screening). About 5–10% of women with risk factors have early GDM, and these represent 40% of all women with GDM.

In the absence of early screening or for women negative to early screen, universal screening should be performed at 24 to 28 weeks.

(c) Now we discuss how to screen.

Screening for GDM is somewhat controversial and can be performed either with a One-Step or with a Two-Step approach.

*(i) One-Step Approach*. GDM screening is performed as an oral 75 g glucose load followed by glucose blood measurement 1 and 2 hours later. A positive result is defined as one value higher than target values. This approach is based on HAPO study [6] and is suggested by IADPSG [7], WHO [8], FIGO [5], and ADA [9]. In fact, HAPO study in 2008 demonstrated a direct correlation between maternal glucose levels and increased birth weight and neonatal hyperinsulinemia.

*(ii) Two-Step Approach*. GDM is performed as a 50 g one-hour oral glucose load (glucose challenge test, GCT), given to nonfasting women, with a venous glucose measurement one hour later. A positive result is defined as a blood glucose value higher than 130, 135, or 140 mg/dL; the most common value used is 135 mg/dL (ACOG) [4]. Positive screening test is followed by a diagnostic test as an oral glucose tolerance test (GTT) that consists of a beverage with 100 g of glucose, with venous glucose measurement at fasting and after 1, 2, and 3 hours. A positive result is defined as 2 values higher than target values.

(d) Recommendations of international organizations are not standardized.

Table 1 shows the different populations and times to screen and the thresholds used by the most important international organizations worldwide, updated to the latest recommendations [4, 5, 8–12].

We found a large number of studies in international literature comparing One-Step and Two-Step test and different glucose thresholds. When evaluating the best screening method, clinically significant improvements in maternal and neonatal outcomes were analyzed. Two are the most significant studies:

1. Australian Carbohydrate Intolerance Study in Pregnant Women (ACHOIS) Trial Group: the aim was to determine whether treatment of GDM reduced the risk of perinatal complications.
2. National Institute of Child Health and Human Development (NICHD) Maternal-Fetal Medicine Units (MFMU) Network Study: the intent was to determine whether treatment of women with mild GDM reduces perinatal and obstetrical complications.

Both trials agree with the rule of IADPSG criteria adoption on reducing fetal birth weight over the 90th percentile and the risk of developing maternal preeclampsia.

Furthermore, to compare the One-Step test to the Two-Step test, several possible study designs have been evaluated in the literature. We can summarize the literature in five groups.

(1) RCTs in which women underwent both the One-Step and the Two-Step tests and the women positive for the One-Step but negative for the Two-Step test were randomized to treatment of GDM versus no treatment: the only such RCT is by Weiss et al. [12], who unfortunately do not report outcomes specific to this group of women.

(2) RCTs of treatment versus no treatment of GDM, focusing on women positive for the One-Step but negative for the Two-Step test: we found 6 RCTs comparing insulin or glyburide to placebo or routine care, and all of them used a Two-Step approach with different glucose thresholds (Table 2) [13–18]. In this group, ACHOIS trial by Crowther et al. [16] is included, mentioned before. The main common outcome was lower rate of fetal birth weight over the 90th percentile and macrosomia.

(3) RCTs comparing the One-Step to the Two-Step methods: we found 3 RCTs by Meltzer et al. [19], Sevket et al. [20], and Scifres et al. [21] (Table 3). In each one, there are a study group undergoing One-Step 75 g test and a control group undergoing Two-Step 100 g test. Regarding GDM rate, Sevket et al.'s and Scifres et al.'s RCTs reveal an incidence more than double in the study group with respect to control group (14.5% versus 6%; 4.3% versus 0.0%), while in Meltzer et al.'s RCT, there are no differences (3.6% versus 3.7%). Maternal and neonatal outcomes have been analyzed only in 2 studies. Sevket et al.'s RCT reveals that GDM-negative women by IADPSG had better perinatal outcomes than GCT-negative women and GCT-positive women with a negative OGTT; Scifres et al.'s RCT concludes that rates of macrosomia, cesarean delivery, and pregnancy-induced hypertension were also similar between groups.

Interestingly, Meltzer et al.'s RCT analyzed costs of the One-Step compared to the Two-Step test: while the Two-Step test involved the lowest costs, the One-Step test recognized higher GDM rate. The authors' conclusion was in favor of the Two-Step test because the universal glucose screen with 50 g glucose load is an inexpensive, easy-to-administer tool for GDM screening, especially with the use of a lower diagnostic cut-off.

(4) Prospective non-RCTs or retrospective studies comparing incidence of GDM and/or outcomes between the One-Step and Two-Step methods: we found 9 retrospective studies comparing the One-Step approach with IADPSG criteria and Two-Step approach with ACOG criteria (Table 4) [22–30]. Regarding GDM rate, the incidence is higher for women undergoing the One-Step test in all the studies analyzing this issue. Only two studies concluded that IADPSG One-Step approach is useful to avoid worse pregnancy outcomes, in particular LGA and macrosomia [22, 27], while five studies did not find statistically significant differences between the two approaches on outcomes [23–26, 28].

(5) Prospective non-RCTs or retrospective studies reporting outcomes of women meeting criteria for GDM based on the One-Step test but not on the Two-Step test: we found 8 retrospective cohort studies (Tables 5 and 6) [31–38], but no study evaluated whether treatment of women meeting criteria for GDM by IADPSG criteria (One-Step test) but not by other less strict criteria has an effect on adverse pregnancy outcomes compared to no treatment. When analyzing outcomes, macrosomia was more common in women positive on 75 g IADPSG criteria but negative on CDA criteria and positive on 75 g IADPSG criteria but negative on NICE criteria.

## 3. Conclusion

Despite continuing controversy regarding whether the One-Step test or the Two-Step test should be used for GDM screening, we identified very limited evidence regarding whether treatment of women meeting criteria for GDM by IADPSG criteria (One-Step test) but not by other less strict criteria has an effect on adverse pregnancy outcomes compared to no treatment. Moreover, in none of the included studies was the study group with milder disease treated for GDM (positive for IADPSG criteria, but negative for less stringent criteria). We also found a large variety of different criteria (IADPSG, WHO, NICE, CDA, and C&C) for screening for GDM used in the literature. Therefore, it is not surprising that societies such as IADPSG, WHO, and FIGO recommend the One-Step approach (assuming that identification of women with milder GDM might have benefits for them and their babies), while others such as ACOG still recommend the Two-Step approach for screening.

Only well designed RCTs comparing the One-Step versus the Two-Step approach including huge populations could answer this question.

## 4. Management Controversies

The aim of management is to reduce the risk of adverse outcomes for the mother and the fetus. Several studies demonstrated that treatment can be effective in reducing adverse outcomes in GDM patients.

Regarding the effect of management on obstetrical outcomes, there are many variables that can play a role; these include

- criteria to start therapy after diet alone: once GDM has been diagnosed, patients start nonpharmacological therapy, that is, well balanced diet based on BMI and physical exercise, but it is unclear how long this evaluation period should last before deciding to start pharmacological treatment; a recent systematic review found inconclusive evidence for the threshold value to start medical therapy [4];
- type of initial therapy: insulin and oral hypoglycemic agents are equally effective and can be used as first-line therapy [5];
- dose and frequency of initial therapy: therapy should start at the lower effective dose and then increase based on glucose monitoring;
- frequency of glucose monitoring: when patients start therapy, either diet or pharmacological therapy is important to establish whether glycemic control has been reached; while patients in pharmacological therapy should perform glycemic checks at least four times daily (fasting and after 1 or 2 hours from three main meals: breakfast, lunch, and dinner), there is uncertainty for women in nonpharmacological therapy [5];
- target glucose values: RCTs to identify ideal glycemic targets have not been performed, but ADA and ACOG recommend a threshold of 140 mg/dL at 1 hour postprandially or 120 mg/dL at 2 hours postprandially as glycemic targets to reduce the risk of macrosomia [5, 9];
- criteria for pharmacologic therapy dose adjustment: when choosing between tight versus very tight glycemic control, we have to consider risk of hypoglycemia, effects of non-well-controlled GDM, and women compliance;
- criteria for adding or switching pharmacologic therapy;
- fetal monitoring;
- time to delivery: women with GDM with good glycemic control and no other complications can be managed expectantly, while if GDM is not well controlled with therapy, induction of delivery could be considered [5].

We analyzed the literature to figure out which management is the best to follow. When evaluating RCTs [16, 17, 39–51] which included criteria for starting pharmacologic therapy in women with GDM, the most common frequency for glucose monitoring was four times per day (i.e., when fasting and after each main meal). The effect of therapy on GDM was assessed using fasting of 90 (or 95) mg/dL and 2 hours of 120 mg/dL as blood glucose target values. Importantly, we found several different criteria for starting pharmacologic therapy after a period of diet alone, with the majority using very tight criteria of either 1 or 2 values in one- or two-week period higher than the target values, of which half used only 1 value and half used 2 values, while any RCT used less tight criteria (i.e., >50% glucose values higher than target values) (Table 7) [16, 17, 39–51].

Finally, when analyzing international organizations guidelines on management of GDM, while there is consensus about glycemic targets, we found different opinions about therapy, monitoring, and time of delivery (Table 8). Moreover, there is limited information regarding other important criteria about dose and frequency of therapy, dose adjustment, and adding or switching pharmacologic therapy.

Moreover, the application of the IADPSG was associated with an increase in GDM prevalence up to 3.5-fold, as well as significant improvements in pregnancy outcomes (gestational hypertension, prematurity, CD, number of LGA and SGA, and 1-minute Apgar scores <7), and was cost-effective. This could be presumably by permitting the treatment of a greater number of women at risk for pregnancy complications [22].

## 5. Conclusion

There are many unsolved questions concerning GDM management. Analyzing the literature in detail, we found different criteria for screening for GDM, for monitoring GDM, and for starting pharmacological therapy. The hope is to reach universally approved and shared recommendations to improve health care and reduce costs and adverse outcomes for women with GDM and their babies.

## Figures and Tables

**Table 1:** Criteria for GDM screening and diagnosis.

	Population to screen	Time to screen	Test	Number of abnormal values required for diagnosis	Fasting glucose (mg/dL)	1 hour after loading (mg/dL)	2 hours after loading (mg/dL)	3 hours after loading (mg/dL)
ACOG 2013 C&C [4]	Selective screening	First visit	Two-Step, 3 h, 100 g	≥2	95	180	155	140
ACOG 2013 NDDG [4]	Selective screening	First visit	Two-Step, 3 h, 100 g	≥2	105	190	165	145
ADA 2015 [9]	Universal screening	24–28 weeks	One-Step, 2 h, 75 g	≥2	95	180	155	Not required
ADA 2015 [9]	Universal screening	First visit	Two-Step, 3 h, 100 g	≥2	95	180	155	140
ADIPS 2013 [52]	Selective screening	24–28 weeks	One-Step, 2 h, 75 g	≥1	92	180	153	Not required
CDA 2013 [10]	Universal screening	First visit	Two-Step, 2 h, 75 g	≥2	95	191	160	Not required
FIGO 2013 [5]	Universal screening	24–28 weeks	One-Step, 2 h, 75 g	≥1	92	180	153	Not required
IADPSG 2010 [7]	Universal screening	24–28 weeks	One-Step, 2 h, 75 g	≥1	92	180	153	Not required
NICE 2015 [11]	Selective screening	24–28 weeks	One-Step, 2 h, 75 g	≥1	101	Not required	140	Not required
WHO 2013 [8]	Universal screening	24–28 weeks	One-Step, 2 h, 75 g	≥1	92	180	153	Not required

ACOG: American College of Obstetricians and Gynecologists; ADA: American Diabetes Association; ADIPS: Australasian Diabetes in Pregnancy Society; CDA: Canadian Diabetes Association; C&C: Carpenter and Coustan; FIGO: International Federation of Gynecology and Obstetrics; IADPSG: International Association of Diabetes Pregnancy Study Group; NICE: National Institute for Health and Care Excellence; NDDG: National Diabetes Data Group; WHO: World Health Organization.

**Table 2:** RCTs of treatment versus no treatment of GDM, focusing on women positive for the One-Step but negative for the Two-Step test.

Study	Screening test	Diagnostic test	Values for diagnosis	Intervention group	Control group	Primary outcome
O'Sullivan et al., 1966 (USA) [13]	50 g GCT: positive if ≥**130** mg/dl	100 g, 3 h (110-170-120-110)	2 or more values	Insulin	Routine care	LGA
Coustan and Lewis, 1978 (USA) [14]	50 g GCT: positive if ≥**130** mg/dl	100 g, 3 h (95-180-160-135)	2 or more values	Insulin	Routine care	Macrosomia
Thompson et al., 1990 (USA) [15]	50 g GCT: positive if F > 105 mg/dL or 1 h > **140** mg/dL	100 g, 3 h (105-190-165-145)	2 or more values	Insulin	Routine care	Maternal and neonatal morbidity
Crowther et al., 2005 (Australia) [16]	50 g GCT: positive if ≥**140** mg/dl	75 g OGTT (F > 7.8; 2 h 7.8–10 mmol/L)	Both values	Insulin	Routine care	Perinatal complications
Landon et al., 2009 (USA) [17]	50 g GCT: positive if ≥**135** mg/dl	100 g, 3 h (95-180-155-140)	2 or more values but F < 95 mg/dL	Insulin	Routine care	Perinatal outcome
Casey et al., 2015 (USA) [18]	50 g GCT: positive if ≥**140** mg/dL	100 g, 3 h (105-190-165-145)	2 values	Glyburide	Placebo	Birth weight

**Table 3:** RCTs comparing the One-Step to the Two-Step methods.

Author (origin)	Study group	Control group (1)	Control group (2)	GDM rate	Primary outcome
Meltzer et al., 2010 (Canada) [19]	One-Step (2 h, 75 g)	Two-Step (50 g, 1 h; 100 g, 3 h)	Two-Step (50 g, 1 h; 75 g, 3 h)	3.6% versus 3.7% versus 3.7%	Costs of screening
Sevket et al., 2013 (Turkey) [20]	One-Step (2 h, 75 g)	Two-Step (50 g, 1 h; 100 g, 3 h)		14.5% versus 6%	Maternal and neonatal outcomes
Scifres et al., 2014 (USA) [21]	One-Step (2 h, 75 g)	Two-Step (50 g, 1 h; 100 g, 3 h)		4.3% versus 0.0%	Maternal and neonatal outcomes

**Table 4:** Prospective non-RCTs or retrospective studies comparing incidence of GDM and/or outcomes between the One-Step and Two-Step methods.

Author (origin)	Study design	Two-Step group	One-Step group	GDM rate	Primary outcome
Duran et al., 2014 (Spain) [22]	Retroprospective cohort	ACOG: 50 g 1 h GCT; if >**140** mg/dL followed by 100 g 3 h GTT (C&C)	IADPSG: 75 g 2 h GTT	10.6% versus 35.5%	Pregnancy outcomes
Fuller and Borgida, 2014 (USA) [23]	Retroprospective cohort	ACOG: 50 g 1 h GCT; if >**135** mg/dL followed by 100 g 3 h GTT (C&C)	IADPSG: 75 g 2 h GTT	7.0% versus 11.7%	Maternal and delivery outcomes
Liu et al., 2014 (China) [24]	Retrospective cohort	ACOG: 50 g 1 h GCT; if >**140** mg/dL followed by 100 g 3 h GTT (C&C)	IADPSG: 75 g 2 h GTT	7.0% versus 20.4%	Maternal and perinatal outcomes
Oriot et al., 2014 (Belgium) [25]	Retrospective cohort	ACOG: 50 g 1 h GCT; if >**140** mg/dL followed by 100 g 3 h GTT (C&C)	IADPSG: 75 g 2 h GTT	8.0% versus 23.0%	CS, macrosomia
Wei et al., 2014 (China) [26]	Retrospective cohort	ACOG: 50 g 1 h GCT; if >**135** mg/dL followed by 75 g 3 h GTT (NDDG)	IADPSG: 75 g 2 h GTT	18.3% versus 21.0%	CS, macrosomia
Hung and Hsieh, 2015 (Taiwan) [27]	Retrospective cohort	ACOG: 50 g 1 h GCT; if >**140** mg/dL followed by 100 g 3 h GTT (C&C)	IADPSG: 75 g 2 h GTT	4.6% versus 12.4%	Macrosomia, LGA
Kong et al., 2015 (Canada) [28]	Retrospective cohort	ACOG: 50 g 1 h GCT; if >**140** mg/dL followed by 100 g 3 h GTT (C&C)	IADPSG: 75 g 2 h GTT	7.9% versus 9.4%	Maternal and fetal outcomes
Assaf-Balut et al., 2016 (Spain) [29]	Retrospective cohort	ADA: 50 g 1 h GCT; if >**140** mg/dL followed by 100 g 3 h GTT (C&C)	IADPSG: 75 g 2 h GTT	Not stated	Postpartum disorders
Klara Feldman et al., 2016 (USA) [30]	Retroprospective cohort	ACOG: 50 g 1 h GCT; if >**130** mg/dL followed by 100 g 3 h GTT (C&C)	IADPSG: 75 g 2 h GTT if HbA1c < 5.7%	17.0% versus 27.0%	Pregnancy outcomes

**Table 5:** Prospective non-RCT or retrospective studies reporting outcomes of women meeting criteria for GDM based on the One-Step test but not on the Two-Step test.

Author (origin)	Study design	GDM screening	50 g GCT criteria	75 g OGTT criteria	100 g OGTT criteria
Lapolla et al., 2011 (Italy) [31]	Retrospective cohort	*Two-Step: 50 g* 1** **h; if >140 mg/dL: *100 g 3 h GTT*	≥**140 **mg/dL: 100 g 3 h GTT	*Not done*	2 abnormal values of fasting ≥ 95 mg/dL, or 1 h 180 mg/dl; 2 h 155 mg/dL; 3 h 140 mg/dL
Bodmer-Roy et al., 2012 (Canada) [32]	Retrospective cohort	*Two-Step: 50 g* 1 h; if 137–184 mg/dL: *75 g 2 h GTT*	137–184 mg/dL: 75 g GTT; >184 mg/dL: GDM	1 abnormal value of fasting ≥ 96 mg/dL; 1 h: ≥191 mg/dl; 2 h: ≥160 mg/dL^*∗*^	*Not done*
Benhalima et al., 2013 (Belgium) [33]	Retrospective cohort	*Two-Step: 50 g* 1 h; if ≥140 mg/dL:*100 g 3 h GTT*	≥**140** mg/dL: 100 g 3 h GTT	*Not done*	2 abnormal values of fasting ≥ 95 mg/dL, or 1 h 180 mg/dl; 2 h 155 mg/dL; 3 h 140 mg/dL
Ethridge et al., 2014 (USA) [34]	Retrospective cohort	*Two-Step: 50 g* 1 h; if ≥135 mg/dL:*100 g 3 h GTT*	≥**135** mg/dL: 100 g 3 h GTT	*Not done*	2 abnormal values of fasting ≥ 95 mg/dL, or 1 h 180 mg/dl; 2 h 155 mg/dL; 3 h 140 mg/dL
Liao et al., 2014 (China) [35]	Retrospective cohort	*Two-Step: 50 g* 1 h; if ≥140 mg/dL: *100 g 3 h GTT*	≥**140** mg/dL: 100 g 3 h GTT	*Not done*	2 abnormal values of fasting ≥ 95 mg/dL, or 1 h 180 mg/dl; 2 h 155 mg/dL; 3 h 140 mg/dL
Mayo et al., 2015 (Canada) [36]	Retrospective cohort	*Two-Step: 50 g* 1 h; if 140–184 mg/dL: *75 g 2 h GTT*	If 140–184 mg/dL: 75 g GTT; >184 mg/dL: GDM	1 abnormal value of fasting ≥ 95 mg/dL; 1 h: ≥191 mg/dl: 2 h: ≥160 mg/dL^*∗*^	*Not done*
Meek et al., 2015 (UK) [37]	Retrospective cohort	*Two-Step: 50 g* 1 h; if >138 mg/dL: *75 g 2 h GTT*	>**138** mg/dL: 75 g 2 h GTT	1 abnormal value of fasting ≥ 110/128 mg/dL; 2 h: ≥140 mg/dL^*∗∗*^	*Not done*
Tward et al., 2016 (Canada) [38]	Retrospective cohort	*Two-Step: 50 g* 1 h; if >140 mg/dL: *75 g 2 h GTT*	≥**140** mg/dL: 75 g 2 h GTT	2 abnormal values of fasting ≥ 95 mg/dL; 1 h: ≥191 mg/dl: 2 h: ≥160 mg/dL	*Not done*

^*∗*^2008 Canadian Diabetes Association criteria (ref.). ^*∗∗*^WHO 1999 criteria until 2007 (fasting, 148 mg/dL), modified WHO 1999 criteria (fasting, 130 mg/dL).

**Table 6:** Continues on the same studies as in Table 5.

Author (origin)	Study group	Control	Primary outcome
Lapolla et al., 2011 (Italy) [31]	*100 g IADPSG-positive, C&C-negative* (fasting: 92–94** **mg/dL; 2** **h: 153-154** **mg/dL; *not treated*) [*n* = 112]	*IADPSG-negative* (fasting: <92 mg/dL; 1 h: <180 mg/dL; 2 h: <153 mg/dL) [*n* = 1815]	Perinatal outcomes

Bodmer-Roy et al., 2012 (Canada) [32]	*75 g IADPSG-positive, CDA-negative* (fasting: 92–95** **mg/dL; 1** **h: 180–190 mg/dL; 2 h: 153–159 mg/dL; *not treated*) [*n* = 186]	*GCT-negative *(50 g 1 h < 137 mg/dL) [*n* = 186] Or *IADPSG-negative* (fasting: <92 mg/dL; 1 h: <180 mg/dL; 2 h: <153 mg/dL) [*n* = 186]	LGA > 90th percentile

Benhalima et al., 2013 (Belgium) [33]	*100 g IADPSG-positive, C&C-negative* (fasting: 92–94 mg/dL; 2 h: 153-154 mg/dL; *not treated*) [*n* = 160]	*GCT-negative *(50 g 1 h < 140 mg/dL) And *IADPSG-negative* (fasting: <92 mg/dL; 1 h: <180 mg/dL; 2 h: <153 mg/dL) [*n* = 6345]	Pregnancy outcomes

Ethridge et al., 2014 (USA) [34]	*100 g IADPSG-positive, C&C-negative* (fasting: 92–94 mg/dL; 2 h: 153-154 mg/dL; *not treated*) [*n* = 281]	*GCT-negative *(50 g 1 h < 135 mg/dL) [*n* = 6999] Or *IADPSG-negative* (fasting: <92 mg/dL; 1 h: <180 mg/dL; 2 h: <153 mg/dL) [*n* = 772]	Birth weight and neonatal outcomes

Liao et al., 2014 (China) [35]	*100 g IADPSG-positive, C&C-negative* (fasting: 92–94 mg/dL; 2 h: 153-154 mg/dL; *not treated*) [*n* = 1314]	*GCT-negative *(50 g 1 h < 140 mg/dL) And *IADPSG-negative* (fasting: <92 mg/dL; 1 h: <180 mg/dL; 2 h: <153 mg/dL) [*n* = 2662]	Maternal and neonatal outcomes

Mayo et al., 2015 (Canada) [36]	*75 g IADPSG-positive, CDA-negative* (fasting: 92–95 mg/dL; 1 h: 180–190 mg/dL; 2 h: 153–159 mg/dL; *not treated*) [*n* = 155]	*GCT-negative* (50 g 1 h < 140 mg/dL) [*n* = 4183] Or *IADPSG-negative* (fasting: <92 mg/dL; 1 h: <180 mg/dL; 2 h: <153 mg/dL) [*n* = 526]	Not stated

Meek et al., 2015 (USA) [37]	*75 g IADPSG-positive, NICE-negative* (fasting: 92–101 mg/dL; 1 h: ≥153 mg/dL; *not treated*) [*n* = 387]	*IADPSG-negative* (fasting: <92 mg/dL; 1 h: <180 mg/dL; 2 h: <153 mg/dL) [*n* = 2406]	Delivery and neonatal outcomes

Tward et al., 2016 (Canada) [38]	*75 g IADPSG-positive, CDA-negative* (fasting: 92–95 mg/dL; 1 h: 180–190 mg/dL; 2 h: 153–159 mg/dL; *not treated*) [*n* = 99]	*GCT-negative* (50 g 1 h < 140 mg/dL) [*n* = 1021] Or *IADPSG-negative* (fasting: <92 mg/dL; 1 h: <180 mg/dL; 2 h: <153 mg/dL) [*n* = 184]	Fetal growth in twins

**Table 7:** Management of women included in RCTs.

	Glucose monitoring	Target value for glycemic control	Type of diet	Recommendations about exercise	Glucose values used for starting pharmacologic therapy based on target values
Garner et al., 1997 [39]	4 times daily^A^	F: <4.4 mmol/l (80 mg/dL); 1 h: <7.8 mmol/l (140 mg/dL)	35 kcal/kg IBW/day	Not stated	2 or more values higher in 2 weeks

Langer et al., 2000 [40]	7 times daily^B^	F: <5.0 mmol/l (90 mg/dL); preprandial: <5.3 mmol/l (95 mg/dl) 2 h: <6.7 mmol/l (120 mg/dL)	(i) 25 kcal/kg BW/day for obese women (ii) 35 kcal/kg BW/day for nonobese women (iii) 3 meals and 4 snacks (iv) 40–45% of calories from carbohydrates	Not stated	1 or more preprandial or 2 h values higher in 1 week

Mecacci et al., 2003 [41]	9 times daily^C^	F: <5.0 mmol/l (90 mg/dL); 1 h: <6.7 mmol/l (120 mg/dL)	ADA recommendations^*∗*^	Not stated	More than 50% values higher after 1 week

Schaefer-Graf et al., 2004 [42]	6 times daily^D^	Intervention group: F: <4.5 mmol/l (80 mg/dL); 1 h: <6.1 mmol/l (110 mg/dL) Control group: F: <5.0 mmol/l (90 mg/dL); 1 h: <6.7 mmol/l (120 mg/dL)	(i) 25 kcal/kg BW/day for overweight women (ii) 30 kcal/kg BW/day for normal weight women	Exercise after meals	Intervention group: (i) AC > 75th *p* < 36 weeks (ii) F ≥ 120 mg/dL (iii) 2 h ≥ 200 mg/dL Control group: (iv) 2 or more values (v) 4 profiles with at least 1 value higher in 2 weeks

Crowther et al., 2005 [16]	4 times daily^E^	F: <5.5 mmol/l (99 mg/dL); 2 h: <7.0 mmol/l (126 mg/dL)	Dietary advice from a qualified dietician	Not stated	(i) 2 values higher in 2 weeks <35 weeks (ii) 2 h >8.0 mmol/l (144 mg/dl) in 2 weeks >35 weeks (iii) 1 value >9.0 mmol/l (162 mg/dl) in 2 weeks

Anjalakshi et al., 2007 [43]	Not specified	2 h: <6.7 mmol/l (120 mg/dL)	Medical Nutrition Therapy (MNT)	Not stated	1 value 2 h higher in 2 weeks

Landon et al., 2009 [17]	4 times daily^E^	F: <5.3 mmol/l (95 mg/dL); 2 h: <6.7 mmol/l (120 mg/dL)	ADA recommendations^*∗∗*^	Not stated	(i) >50% values higher between 2 study visits (ii) 1 random value >160 mg/dl (8.9 mmol/l) (iii) 1 F > 95 mg/dl; the patient's caregiver initiated treatment (more or less 7 visits)

Ijäs et al., 2011 [44]	4 times daily^F^	F: <5.3 mmol/l (95 mg/dL); 1.5 h: <6.7 mmol/l (120 mg/dL)	Dietary and lifestyle counselling	Not stated	2 values higher in 2–4 weeks

Balaji et al., 2012 [45]	4 times daily^E^	F: <5.0 mmol/l (90 mg/dL); 2 h: <6.7 mmol/l (120 mg/dL); HbA1c: <6.0 g/dL	Medical Nutrition Therapy (MNT)	Not stated	1 value higher in 2 weeks

Mukhopadhyay et al., 2012 [46]	7 times daily^B^	F: <5.0 mmol/l (90 mg/dL); 2 h: <6.7 mmol/l (120 mg/dL)	(i) 25 kcal/kg BW for obese women (ii) 35 kcal/kg BW for nonobese women (iii) 3 daily meals; 40–45% of calories from carbohydrates	Not stated	1 value higher in 2 weeks

Niromanesh et al., 2012 [47]	4 times daily^E^	F: <5.3 mmol/l (95 mg/dL); 2 h: <6.7 mmol/l (120 mg/dL)	(i) 15 kcal/kg BW for obese women (ii) 22 kcal/kg BW for overweight women (iii) 30 kcal/kg BW for normal weight women (iv) 40 kcal/kg BW for underweight women (v) 45% of calories from carbohydrates, 20% from protein, and 35% from fat (vi) 3 meals and 3 snacks (vii) Calories: 10% breakfast, 30% each lunch and dinner, and 30% snacks	30 minutes of walking per day	2 values higher in one week

Silva et al., 2010 [48]	4 times daily^A^	F: <5.0 mmol/l (90 mg/dL); 1 h: <6.7 mmol/l (120 mg/dL)	(i) 25 kcal/kg BW/day for overweight women (ii) 35 kcal/kg BW/day for normal weight women (iii) 3 full meals and 4 light meals (iv) 35–45% of calories from carbohydrates	Not stated	2 values higher after 1 week

Mesdaghinia et al., 2013 [49]	4 times daily^E^	F: <5.3 mmol/l (95 mg/dL); 2 h: <6.7 mmol/l (120 mg/dL)	Dietary changes^*∗∗∗*^	Not stated	1 value higher in 1 week

Spaulonci et al., 2013 [50]	4 times daily^E^	F: <5.3 mmol/l (95 mg/dL); 2 h: <6.7 mmol/l (120 mg/dL)	(i) 25–35 kcal/kg IBW based on pregestational BMI (ii) 55% carbohydrates, 15% proteins, and 30% fat	30-minute walk 3 times a week	>30% values higher in 1 week

Behrashi et al., 2016 [53]	4 times daily^E^	F: <5.0 mmol/l (90 mg/dL); 2 h: <6.7 mmol/l (120 mg/dL)	Education for lifestyle change (exercise and diet)	Education for lifestyle change (exercise and diet)	1 value higher in 1 week

F: fasting; GA: gestational age; IBW: ideal body weight; BW: body weight; BMI: body mass index.

^A^Fasting and 1 hour after each main meal: breakfast, lunch, and dinner.

^B^Fasting, before lunch and dinner, 2 hours after main meals, breakfast, lunch, and dinner, and at bedtime.

^C^Fasting, preprandial before lunch and dinner, 1 and 2 hours after each main meal: breakfast, lunch, and dinner.

^D^Fasting, preprandial before lunch and dinner, 1 hour after each main meal: breakfast, lunch, and dinner.

^E^Fasting and 2 hours after each main meal: breakfast, lunch, and dinner.

^F^Fasting and 1.5 hours after each main meal: breakfast, lunch, and dinner.

^*∗*^American Diabetes Association, Medical Management of Pregnancy Complicated by Diabetes, 3rd Edition, Alexandria, Virginia; ADA, 2000, pp. 70–86.

^*∗∗*^American Diabetes Association, Nutrition Recommendations and Interventions for Diabetes: A Position Statement of the American Diabetes Association; Diabetes Care 2008 Jan. 31 (Suppl. 1): S61–S78.

^*∗∗∗*^Cheung NW, The Management of Gestational Diabetes: A Review Article; Vasc Health Risk Manag. 2009; 5:153–64.

**Table 8:** Management of GDM, international guidelines.

	ACOG 2013 [4]	CDA 2013 [10]	ADA 2015 [9]	FIGO 2015 [5]	NICE 2015 [11]
*Criteria to start therapy after diet alone*	Inconclusive evidence	Glycemic control not achieved after 2 weeks of nutritional therapy alone	NR	NR	Glycemic control not achieved after 1-2 weeks of diet and exercise

*Type of initial therapy*	Insulin or oral medications	Insulin or oral medications	Insulin or glyburide	Glyburide inferior to both insulin and metformin, while metformin performs better than insulin	Metformin

*Dose and frequency of initial therapy*	NR	NR	NR	NR	NR

*Frequency of glucose monitoring*	4 times daily as fasting and either 1 h or 2 h after each meal	4 times daily as fasting and either 1 h or 2 h after each meal	NR	4 times daily as fasting and 2 h after each meal	7 times daily as fasting, premeal, 1 h after each meal, bedtime

*Target glucose values*	1 h ≤ 140 mg/dL, 2 h ≤ 120 mg/dL	Fasting ≤ 95 mg/dL, 1 h ≤ 140 mg/dL, 2 h ≤ 120 mg/dL	Fasting ≤ 95 mg/dL, 1 h ≤ 140 mg/dL, 2 h ≤ 120 mg/dL	Fasting ≤ 95 mg/dL, 1 h ≤ 140 mg/dL, 2 h ≤ 120 mg/dL	Fasting ≤ 95 mg/dL, 1 h ≤ 140 mg/dL, 2 h ≤ 116 mg/dL

*Criteria for pharmacologic therapy dose adjustment*	NR	NR	NR	NR	NR

*Criteria for adding or switching pharmacologic therapy*	NR	NR	NR	NR	NR

*Pregnancy monitoring*	No consensus	NR	NR	NR	Ultrasound monitoring of fetal growth and AF volume every 4 weeks from 28 to 36 weeks

*Time to delivery*	Well-controlled: >39 weeks; insufficient data for others; CD if EFW > 4500 g	NR	NR	Consider induction at 38-39 weeks	Delivery no later than 40 + 6 weeks

NR: not reported.
